# Porro's Operation and Cæsarian Section

**Published:** 1900-07-28

**Authors:** 


### PORRO'S OPERATION AND CÆSARIAN SECTION.

At the last meeting of the Obstetrical Society a paper was read by Dr. Amand Routh which led to an interesting discussion as to the relative advantages of the "Modern Porro," and the various other operations which have been used in place of Cæsarian section. The case which Dr. Amand Routh described was one in which the pelvis was occupied by a large fibroid, and the cervix was out of reach above the symphysis. As it was found that the head was arrested just above the lower segment of the uterus by two opposing fibroids, a Porro-Cæsarian operation was performed in preference to a panhysterectomy, or to Cæsarian section with removal of the appendages (sterilising Cæsarian section). From the position of the tumour it was necessary to make the incision into the uterus directly over the placental site. The placenta was stripped off, the membranes were incised, the child was extracted, the placenta and membranes were removed, and the uterus was brought out through the abdominal incision. The operation was then completed by Baer's method. Both mother and child made an uneventful recovery. It was, said Dr. Routh, a point worthy of discussion, whether supra-vaginal amputation of the uterus (Porro's operation), with retroperitoneal treatment of the stump, was not safer for the patient in skilled hands than a Sänger-Cæsarian operation with sterilisation in all cases of permanent obstruction to labour requiring abdominal section, except, perhaps, those due to cancer of the supra-vaginal cervix. It was agreed, he said, that a Porro or a panhysterectomy was absolutely indicated in the following cases: Obstructing fibroids, cicatricial stenosis of the vagina where the lochia could not escape, septic endometritis, decomposed fœtus, osteomalacia, owing to the fact that removal of the appendages was often curative, uterine hæmorrhage from uterine inertia during Cæsarian section, and after much previous manipulation of the uterus in attempts to extract per vaginam. Sänger-Cæsarian section without sterilisation was on the other hand absolutely indicated instead of a Porro where it was considered desirable for the woman to have a chance of another child; and Sänger-Cæsarian with sterilisation was possibly (?) required in cases of cervical cancer when the supra-vaginal portion was involved. There remained, however, a third group of cases where a Cæsarian section with sterilisation was usually done, but where it would in Dr. Routh's opinion be quite as reasonable to do a modern Porro, that is one in which the stump is covered with peritoneum. This third group included all cases of sufficient pelvic contraction, and it was in such cases that the relative value of a modern Porro operation as compared with a sterilising Sänger-Cæsarian demanded serious consideration. The question, as discussed, appeared to hinge very much upon the propriety of inducing sterility for the future at the time of doing a Cæsarian section, and Dr. Routh received considerable support in his contention that rather than do such an operation it would often be better to do the operation which he described as a modern Porro, but which Dr. Galabin thought might be more properly described as a Cæsarian hysterectomy. Dr. Herbert Spence, however, raised an important point in regard to repeated Cæsarian section. He said that while he did not consider that Porro's operation should be done in all cases of permanent obstruction, such as contracted pelvis, he would have nothing to do with sterilisation by tying or cutting the tubes, and he maintained that the conservative Cæsarian section was a very successful operation. Among its advantages he placed the fact that in consequence of the puerperal uterus lying up against and becoming adherent to the abdominal wall, the abdominal cavity was sometimes not opened at all in a subsequent operation. He had, he said, recently performed Cæsarian section under local anæsthesia upon a patient whom he had delivered by Cæsarian section twice previously, and as both the children thus delivered had subsequently died he thought the advantage to the mother of having a third, and this time a very healthy child was obvious. He said he had also successfully repeated Cæsarian section in another case. It is to be confessed that the prospect of having a family by way of repeated abdominal section is a rather startling development of gynæcological surgery.

